# Supplementary material for: Modeling glioblastoma heterogeneity as a dynamic network of cell states
Source: Mol Syst Biol. 2021 Sep 16;17(9):e10105. doi: 10.15252/msb.202010105 (PMC8444284; doi:10.15252/msb.202010105)
Supplement: Supplementary file 6 — Source Data for Figure 5 [file MSB-17-e10105-s004.zip › Figure5A_sourcedata/GSEA_3017/hallmarks_stateE.GseaPreranked.1621934677158/HALLMARK_IL2_STAT5_SIGNALING.html]

Details for gene set HALLMARK\_IL2\_STAT5\_SIGNALING[GSEA]

|  || Dataset | state63017 |
| Phenotype | NoPhenotypeAvailable |
| Upregulated in class | na\_pos |
| GeneSet | HALLMARK\_IL2\_STAT5\_SIGNALING |
| Enrichment Score (ES) | 0.41767764 |
| Normalized Enrichment Score (NES) | 1.8129494 |
| Nominal p-value | 0.011070111 |
| FDR q-value | 0.012951341 |
| FWER p-Value | 0.029 |
Table: GSEA Results Summary

  

Fig 1: Enrichment plot: HALLMARK\_IL2\_STAT5\_SIGNALING      
 Profile of the Running ES Score & Positions of GeneSet Members on the Rank Ordered List

  

| PROBE | GENE SYMBOL | GENE\_TITLE | RANK IN GENE LIST | RANK METRIC SCORE | RUNNING ES | CORE ENRICHMENT || 1 | CD44 |  |  | 14 | 0.806 | 0.1010 | Yes |
| 2 | IFITM3 |  |  | 26 | 0.682 | 0.1874 | Yes |
| 3 | EMP1 |  |  | 40 | 0.584 | 0.2576 | Yes |
| 4 | WLS |  |  | 65 | 0.472 | 0.2998 | Yes |
| 5 | SERPINB6 |  |  | 77 | 0.440 | 0.3514 | Yes |
| 6 | AHNAK |  |  | 125 | 0.351 | 0.3515 | Yes |
| 7 | RHOB |  |  | 148 | 0.335 | 0.3762 | Yes |
| 8 | CSF1 |  |  | 203 | 0.293 | 0.3604 | Yes |
| 9 | BATF3 |  |  | 211 | 0.288 | 0.3944 | Yes |
| 10 | CKAP4 |  |  | 250 | 0.262 | 0.3914 | Yes |
| 11 | SPP1 |  |  | 261 | 0.257 | 0.4177 | Yes |
| 12 | AHCY |  |  | 317 | -0.262 | 0.3964 | No |
| 13 | CCNE1 |  |  | 571 | -0.344 | 0.1742 | No |
| 14 | GSTO1 |  |  | 731 | -0.459 | 0.0695 | No |
| 15 | PHTF2 |  |  | 775 | -0.523 | 0.0985 | No |
| 16 | CDC6 |  |  | 807 | -0.584 | 0.1493 | No |
Table: GSEA details [plain text format]

  

Fig 2: HALLMARK\_IL2\_STAT5\_SIGNALING: Random ES distribution      
 Gene set null distribution of ES for **HALLMARK\_IL2\_STAT5\_SIGNALING**
